# Systemic chemotherapy of pediatric recurrent ependymomas: results from the German HIT-REZ studies

**DOI:** 10.1007/s11060-021-03867-8

**Published:** 2021-10-16

**Authors:** Jonas E. Adolph, Gudrun Fleischhack, Christine Gaab, Ruth Mikasch, Martin Mynarek, Stefan Rutkowski, Ulrich Schüller, Stefan M. Pfister, Kristian W. Pajtler, Till Milde, Olaf Witt, Brigitte Bison, Monika Warmuth-Metz, Rolf-Dieter Kortmann, Stefan Dietzsch, Torsten Pietsch, Beate Timmermann, Stephan Tippelt, for the German GPOH HIT-Network

**Affiliations:** 1 Department of Pediatrics III, Center for Translational Neuro- and Behavioral Sciences (CTNBS), University Hospital of Essen, Hufelandstraße 55, 45147 Essen, Germany; 2 Department of Pediatric Hematology and Oncology, Center for Obstetrics and Pediatrics, University Medical Center Hamburg-Eppendorf, Hamburg, Germany; 3 Institute of Neuropathology, University Medical Center Hamburg-Eppendorf, Hamburg, Germany; 4 Research Institute Children’s Cancer Center Hamburg, Hamburg, Germany; 5 Hopp Children’s Cancer Center Heidelberg (KiTZ), Heidelberg, Germany; 6 Division of Pediatric Neurooncology, German Cancer Research Center (DKFZ), Heidelberg, Germany; 7 Clinical Cooperation Unit Pediatric Oncology, German Cancer Research Center (DKFZ) and German Consortium for Translational Cancer Research (DKTK), Heidelberg, Germany; 8 Department of Pediatric Oncology and Hematology, Heidelberg University Hospital, Heidelberg, Germany; 9 Diagnostic and Interventional Neuroradiology, University Hospital Augsburg, Augsburg, Germany; 10 Institute of Diagnostic and Interventional Neuroradiology, University of Würzburg, Würzburg, Germany; 11 Department of Radio-Oncology, University of Leipzig, Leipzig, Germany; 12 Institute of Neuropathology, DGNN Brain Tumor Reference Center, University of Bonn, Bonn, Germany; 13 Clinic for Particle Therapy, West German Proton Therapy Center, University of Essen, Essen, Germany

**Keywords:** Ependymoma, Chemotherapy, Recurrence, Children, Sirolimus

## Abstract

**Purpose:**

Survival in recurrent ependymoma (EPN) depends mainly on the extent of resection achieved. When complete resection is not feasible, chemotherapy is often used to extend progression-free and overall survival. However, no consistent effect of chemotherapy on survival has been found in patients with recurrent EPN.

**Methods:**

Systemic chemotherapeutic treatment of 138 patients enrolled in the German HIT-REZ-studies was analyzed. Survival depending on the use of chemotherapy, disease-stabilization rates (RR), duration of response (DOR) and time to progression (TTP) were estimated.

**Results:**

Median age at first recurrence was 7.6 years (IQR: 4.0–13.6). At first recurrence, median PFS and OS were 15.3 (CI 13.3–20.0) and 36.9 months (CI 29.7–53.4), respectively. The Hazard Ratio for the use of chemotherapy in local recurrences in a time-dependent Cox-regression analysis was 0.99 (CI 0.74–1.33). Evaluable responses for 140 applied chemotherapies were analyzed, of which sirolimus showed the best RR (50%) and longest median TTP [11.51 (CI 3.98; 14.0) months] in nine patients, with the strongest impact found when sirolimus was used as a monotherapy. Seven patients with progression-free survival > 12 months after subtotal/no-resection facilitated by chemotherapy were found. No definitive survival advantage for any drug in a specific molecularly defined EPN type was found.

**Conclusion:**

No survival advantage for the general use of chemotherapy in recurrent EPN was found. In cases with incomplete resection, chemotherapy was able to extend survival in individual cases. Sirolimus showed the best RR, DOR and TTP out of all drugs analyzed and may warrant further investigation.

**Supplementary Information:**

The online version contains supplementary material available at 10.1007/s11060-021-03867-8.

## Introduction

Recurrent ependymomas (EPN) in children and adolescents feature a poor prognosis, with a median survival after diagnosis of recurrence of only 12 months [1, 2]. Out of all patients diagnosed with a WHO grade II or III EPN, around 40% experience a progression or a relapse [3, 4].

While survival-benefits for surgery and radiotherapy have been shown in multiple cohorts of recurrent EPN, results on chemotherapy were less favorable [1, 5–8]. So far, no uniform chemotherapeutic treatment regimen for recurrent EPN has been established. Results on the efficacy of individual drugs and specific combinations have been almost entirely restricted to trials with limited case-numbers [9, 10].

With advances in molecular characterization emerging in recent years, molecularly defined types of EPN are increasingly treated as distinct diseases, with the infratentorial PF-A and supratentorial ZFTA subtype being the most aggressive and most abundant in recurrences [11–13]. However, most trials on chemotherapy in recurrent EPN were done prior to these findings. Therefore, results on the efficacy in specific EPN types are still lacking. Recent molecular analyses have also suggested that EPN in general may possess features making them especially resistant to chemotherapy [14, 15], pointing towards the importance of finding chemotherapy-regimens that work specifically for EPN.

Here we report on a pooled cohort of patients with recurrent EPN from the multi-institutional HIT-REZ-studies. We examine the influence of chemotherapy on survival and report on cases in which its use showed advantages. Furthermore, we examine the responses to different chemotherapy regimens and analyze them accounting for specific EPN types.

## Methods

### Clinical trials

The HIT-REZ-studies consisted of two multi-institutional trials [HIT-REZ 97 and HIT-REZ 2005 (NCT00749723)], as well as an ongoing registry (HIT-REZ registry) concerning recurrent CNS-tumors in children. Data was gathered through a centralized reporting system. Information included dates of recurrences, metastatic stage, extent of resection, as well as target volume and doses of radiotherapy.

Tumor response was measured through centralized assessment of MRI. An assessment of the response to chemotherapy could only be made if either at least one lesion was able to be clearly measurable in three dimensions, a non-measurable lesion was present or if malignant cells were detectable in the cerebrospinal fluid (CSF) before chemotherapy was applied. If a preceding surgery led to a complete resection of the tumor, only the time to next progression was measured. If a lesion could be defined, complete remission (CR) was defined as a complete absence of all lesions and malignant cells in CSF after systemic treatment. Partial remission (PR) was defined as a decrease in tumor volume of measurable lesion(s) by at least 50%. Stable diseases (SD) were cases of a decrease in volume of less than 50% and no more than 25% of volume-gain or cases with persistent presence of non-measurable lesions. Progressive disease (PD) was chosen when MRI showed an increase in volume of at least 25% or occurrence of new measurable/non-measurable lesion(s) or new detection of tumor cells in the CSF.

Extent of resection was determined by postoperative MRI, with gross-total resection (GTR) being defined as no visible residual tumor. Near-total resection (NTR) was defined as enhancement at the edge of the resection area and a reduction in volume of at least 90%. Surgery with less reduction in tumor volume was defined as a subtotal resection (STR), or as biopsy if no more than 10% was removed.

### Statistical analysis

Response-rates (RR) were defined as the rate of CR, PR and SD being achieved through chemotherapy, while the objective response-rate (ORR) included only CR and PR. If surgery preceded chemotherapy in case of local or unifocal relapse, the residual tumor had to be measurable in three dimensions by MRI before chemotherapy was started to evaluate response. The duration of response (DOR) was defined as the time from the beginning of chemotherapy until the latest date of imaging in which the best grade of response could be found. If PD was the only response found for a chemotherapy regimen, DOR was set to zero months. The time to progression (TTP) was defined as the time from the start of chemotherapy to the time-point a progression was found on MRI and/or new tumor cells were detected in CSF cytology. Recurrences without measurable tumor residual after surgery were only evaluated as to TTP.

Overall survival (OS) was defined as the time from first recurrence to either death from any cause or to the last time-point of follow-up. Progression-free survival (PFS) was defined as the time to next recurrence, death or last follow-up from prior recurrence. For cases of last follow-up censoring was used. Both OS and PFS were given as a median with its accompanying 95% confidence interval (CI). For descriptive statistics of the study cohort, medians were given with their interquartile ranges (IQR) if not differently specified.

Cox-regressions were used to examine the effect of specific covariates on either PFS or OS, as specified. Time-dependent Cox-regressions were used for covariates subject to change during the studies and the follow-up, with the date of diagnosis of the first recurrence was taken as a starting point. Further recurrences as well as death were treated as cumulative events. Results of these Cox-regressions were calculated as a hazard ratio (HR) towards either OS or PFS and its 95%-CI. P values for the HR were given in the calculation of the efficacy of different drugs depending on the molecular subgroup, with the α-value set at 0.05.

All statistical analyses were done using R version 4.0.3 [16], using the *survival* package for all survival analyses and *ggplot2* to produce all figures. The results section of this paper was compiled using R Markdown [17].

## Results

138 patients with recurrent EPN (WHO grade II or III) from the HIT-REZ studies diagnosed between 1998 and 2018 were included in this analysis. They were followed up for a median of 31.6 months (IQR: 16.7–58.1, range: 2.3–197.4) and for a total number of 335 recurrences. Table 1 displays an overview of the characteristics of the analyzed cohort. Patients were predominantly male (65.2%) and had a median age of 7.6 years (IQR: 4.0–13.6, range: 0.8–28.8) at first recurrence. EPN were mostly of infratentorial origin (74.6%). Molecular classification was available for 64.5% of cases, of which 74.2% were PF-A and 20.2% ST-ZFTA.

**Table 1:** Patient characteristics at first recurrence

Characteristic	N = 138 (%)	
Sex		
Male		90 (65.2%)
Female		48 (34.8%)
Localisation at first diagnosis		
Supratentorial		31 (22.5%)
Infratentorial		103 (74.6%)
Spinal		4 (2.9%)
Histological tumor grade		
WHO °II		13 (9.4%)
WHO °III		125 (90.6%)
Molecular subgroup	89 (64)	
PF-A		66 (74.2%)
PF-B		2 (2.2%)
ZFTA		18 (20.2%)
YAP1		2 (2.2%)
MYCN		1 (1.1%)
Median age at first recurrence; years (IQR)		7.6 (4.0, 13.6)
Median time to first recurrence, months (IQR)		22.8 (14.4, 39.9)
Metastatic stage at first recurrence		
M0		74 (53.6%)
M1		4 (2.9%)
M2		25 (18.1%)
M3		33 (23.9%)
M4		2 (1.4%)
Surgery at first recurrence	105 (76)	
GTR		62 (59.0%)
NTR		32 (30.5%)
STR		8 (7.6%)
Biopsy		3 (2.9%)
Radiotherapy at first recurrence	70 (51)	
CSI		23 (32.9%)
Focal radiotherapy		47 (67.1%)
Median target volume dose of focal radiotherapy; Gy (IQR)	69 (50)	50.4 (46.0, 54.0)
Median target volume dose of CSI; Gy (IQR)	22 (16)	35.2 (35.2, 35.2)
Radiotherapy at initial diagnosis		120 (87.0%)
Median target volume dose at initial diagnosis; Gy (IQR)		59.4 (54.0, 68.0)
Chemotherapy at initial diagnosis		108 (78.3%)
Chemotherapy at 1st recurrence		99 (71.7%)
Chemotherapy at 2nd recurrence		47 (34.1%)
Chemotherapy at 3rd recurrence		29 (21.0%)
Chemotherapy at 4th recurrence		14 (10.1%)
Chemotherapy at 5th recurrence		8 (5.8%)

*IQR* interquartile range, *GTR* gross-total resection, *NTR* near-total resection, *STR* subtotal resection, *CSI* craniospinal irradiation

### Survival

Median OS from first recurrence was 36.9 months (CI 29.7–53.4). Median PFS from first recurrence was 15.3 months (CI 13.3–20.0). To analyze whether chemotherapy had an effect on survival, we used a time-dependent Cox-regression model. The use of chemotherapy in all recurrences was recorded, and their cumulative number taken as time-dependent covariates. This resulted in a HR of 1.73 (CI 1.29–2.32) for the use of chemotherapy. To examine whether this result may have been biased by a more prominent use of chemotherapy in cases with increased therapeutic pressure due to more progressive disease and therefore worse outcome, we limited the model to only include local relapses. In contrast to the initial result, a HR for chemotherapy of 0.99 (CI 0.74–1.33) was found for non-metastatic relapses.

To further examine whether the application of chemotherapy might prolong PFS after resection, we compared the median PFS with or without chemotherapy after either GTR/NTR or STR/no resection at first recurrence. In patients with GTR/NTR (n = 94), those treated with chemotherapy showed a longer median PFS than those who did not receive it [19.8 months (CI 15.1–28.8) vs. 15.1 months (CI 8.8–20.4)]. This also held true when correcting for the use of radiotherapy within the same relapse when comparing chemotherapy vs. no chemotherapy [21.1 months (CI 15.4–33.1) vs. 14 months (CI 8.8–39.8) in patients treated with radiotherapy and 19.3 months (CI 13.8–36.5) vs. 15.1 months (CI 6.2–NA) in patients without further radiotherapy]. In contrast, in the group of patients in whom only STR could be achieved or who underwent no surgery (n = 43), chemotherapy did not improve the median PFS [10.4 months (CI 7.2–16.5)] compared to a small group of seven patients who did not receive chemotherapy and had a median PFS of 20.5 months (CI 9–NA). In this group without complete resection, radiotherapy led to a notable increase in median PFS [17.5 months (CI 12.9–25.5) vs. 4.9 months (CI 3.8–16.5)], while chemotherapy did not improve survival within these subsets of patients.

Time from initial diagnosis to first recurrence severely affected survival after recurrence, as well as RR to chemotherapy applied. Patients in whom the first recurrences occurred over 24 months after first diagnosis showed improved PFS [20.5 (CI 15.3–26.5) vs. 13.3 (CI 9.9–17) months], OS [53.4 (CI 36.9–NA) vs. 24.7 (CI 20.4–43) months] and a higher rate of RR as a mean of all systemic therapies applied (33.5% vs. 18.9%). Additional information on survival is provided within the supplements.

### Chemotherapeutic agents

**Fig. 1:**
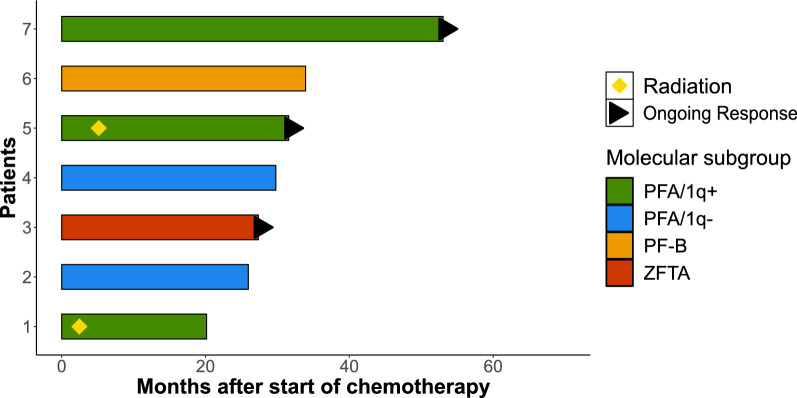
Patients with subtotal or no resection at any recurrence and a PFS of over 12 months. Ongoing response denoted at date of last follow-up. Patient (P) 1: 1st recurrence, Vincristine + Cyclophosphamide + Carboplatin + Etoposide (HIT-SKK); P2: 3rd recurrence, Etoposide + Trofosfamide (HIT-REZ 2005); P3: 1st recurrence, HIT-SKK then Actinomycin D + Etoposide + Trofosfamide; P4: 5th recurrence, 5-FU; P5: 1st recurrence, Etoposide + Trofosfamide (HIT-REZ 2005); P6: 1st recurrence, Temozolomide, P7: 2nd recurrence, Temozolomide (HIT-REZ 2005)

In total, 40 different chemotherapeutic drugs were used in 59 separate single-drug applications or combinations. Evaluable responses could be ascertained for 140 of 236 applied chemotherapies. Table 2 shows all drugs used in at least five treatment courses which were eligible for measurement of response. The most commonly used single-drug chemotherapy was temozolomide applied as in the E-HIT-REZ 2005 protocol with 37 applications, however a RR of only 10.8% was found, with a median DOR of 0 months (CI 0; 0) and a median TTP of 2.56 months (CI 1.58; 4.7). A patient with a PF-A subtype EPN treated with monotherapeutic Temozolomide for 21 months according to the E-HIT-REZ-2005 protocol was the only patient in our cohort to in whom CR by chemotherapy alone was achieved, lasting for five years at current follow-up (*Patient 7 in* Fig. 1). Across all combinations of chemotherapy, etoposide was used most often (54 times), with a RR of 38.9% [median DOR: 0 months (CI 0; 5.04)], median TTP: 3.54 months (CI 1.64; 12.45). Out of all drugs used at least five separate times, sirolimus showed the best RR (50%), the longest median DOR [1.28 months (CI 0; 5.95)] and median TTP [11.51 months (CI 4.22; 14)] across all combinations applied in nine patients. Interestingly, sirolimus seemed to show the best response when used as a monotherapy [RR = 50%, median DOR = 2.46 months (CI 0; 6.29) and median TTP = 14.52 months (CI 10.65; 17.68)]. All patients treated with sirolimus had an EPN of infratentorial origin (7 PFA, 1 ZFTA, 1 unknown molecular type), data for all patients are shown in Table 3.

**Table 2:** Eight most frequently used chemotherapy drugs with evaluable responses

Drug	n	CR	PR	SD	PD	ORR (%)	RR (%)	Median duration of response	Median time to progression
Etoposide	54	0	3	18	33	5.6	38.9	0 (CI 0; 5.04)	3.54 (CI 1.64; 12.45)
Temozolomide	46	1	3	9	33	8.7	28.3	0 (CI 0; 0.79)	2.64 (CI 1.59; 8.09)
Trofosfamide	30	0	1	10	19	3.3	36.7	0 (CI 0; 6.13)	4 (CI 1.81; 11.44)
Carboplatin	20	0	1	7	12	5	40	0 (CI 0; 4.69)	3.93 (CI 1.71; 12.87)
Cyclophosphamide	13	0	0	5	8	0	38.5	0 (CI 0; 2.24)	3.17 (CI 1.93; 8.97)
Vincristine	12	0	0	4	8	0	33.3	0 (CI 0; 2.64)	3.29 (CI 2.2; 7.46)
Sirolimus	8	0	0	4	4	0	50	1.28 (CI 0; 5.95)	11.51 (CI 4.22; 14)
Topotecan	7	0	0	0	7	0	0	0 (CI 0; 0)	1.32 (CI 0.55; 1.61)

*n* Number of times used*, CR* complete remission*, PR* partial remission*, SD* disease stabilization*, PD* progressive disease*, ORR* objective response-rate*, RR* response-rate*, CI* 95% confidence-interval

**Table 3:** List of all patients treated with Sirolimus

Patient	Sex	Age at first recurrence (years)	Initial tumor site	Molecular subgroup	Recurrence number at Sirolimus application	Combination partner(s)	Resection at same recurrence	Radiation at same recurrence	Best response	Time to progression (months)
*Evaluable responses*										
1	Male	3.4	Infratentorial	*NA*	4	+ Imatinib, Topotecan, Temozolomide	NTR	No	PD	1.3
2	Male	3.6	Infratentorial	PF-A	2	+ Dasatinib, Irinotecan, Temozolomide	NTR	Yes	SD	15.3
3	Male	3.1	Infratentorial	PF-A	3	–	No resection	Yes	SD	24.0
4	Female	8.9	Infratentorial	*NA*	3	+ Dasatinib, Irinotecan, Temozolomide	STR	Yes	PD	4.9
5	Male	10.1	Infratentorial	PF-A	3	–	No resection	Yes	PD	13.5
					4	–	STR	No	PD	2.3
6	Female	2.8	Infratentorial	PF-A	2	+ Dasatinib, Irinotecan, Temozolomide	No resection	Yes	SD	13.5
7	Male	0.9	Infratentorial	ZFTA	3	–	No resection	Yes	SD	15.6
*Applications after surgery without evaluable rest-tumor*										
7	Male	0.9	Infratentorial	ZFTA	4	–	GTR	No	n.e	40.2+
8	Male	4.5	Infratentorial	PF-A	1	+ Sunitinib, Irinotecan, Temozolomide	GTR	No	n.e	5.8
9	Male	3.3	Infratentorial	PF-A	1	–	GTR	Yes	n.e	53.6

Best response could only be evaluated if previous to response measurement no surgery was performed or enough rest-tumor was present after surgery to measure the response to the applied chemotherapy

*GTR* gross-total resection, *NTR* near-total resection, *STR* sub-total resection, *SD* stable disease, *PD* progressive disease, *n.e.* not evaluable

+ indicates ongoing response at last follow-up

### Chemotherapy combinations

Our analysis showed 31 combinations of at least two drugs. However, only a small fraction of three combinations were used at least five times. The three most commonly used protocols were E-HIT-REZ 2005 (second line treatment of etoposide + trofosfamide), HIT-SKK (in chemotherapy-naive patients only; carboplatin + cyclophosphamide + etoposide + vincristine) and HIT-REZ 97 (carboplatin + etoposide). The HIT-REZ 97 protocol showed the best RR (44.4%) and TTP (8.93 (CI 3.78; 14.22) months) out of these three.

### Chemotherapy in different molecular types

To examine whether any chemotherapeutic drug showed an improved efficacy on OS in specific molecular EPN types, we calculated HRs regarding OS, depending on whether a specific chemotherapeutic drug was given to a patient or not during treatment of all recurrences. HR were calculated for all drugs used in at least ten patients. Table 4 lists the three drugs with the lowest HR in all patients and in the four most common types. No significant advantage for a specific drug was found in any molecular type. However, trofosfamide and sirolimus showed a trend towards being more efficacious in PF-A tumors with or without chromosome 1q-gain, respectively. Overall, temozolomide and sirolimus showed the most improved HRs observed across all types of EPN.

**Table 4:** Cox-regression comparing OS depending on whether or not a patient received specific chemotherapy drugs

Subgroup	Chemotherapy	HR	95%-CI	p value
All	Temozolomide	0.7	(0.5–1.1)	0.1
	Sirolimus	0.7	(0.3–1.7)	0.44
	Vincristine	0.97	(0.5–1.7)	0.93
PFA	Temozolomide	0.71	(0.4–1.4)	0.31
	Sirolimus	0.85	(0.3–2.4)	0.75
	Trofosfamide	0.89	(0.5–1.7)	0.72
PFA/1q+	Trofosfamide	0.6	(0.2–1.7)	0.34
	Cyclophosphamide	0.7	(0.2–2.4)	0.57
	Temozolomide	0.85	(0.3–2.2)	0.74
PFA/1q−	Sirolimus	0.39	(0.1–3)	0.36
	Temozolomide	0.61	(0.2–1.6)	0.31
	Trofosfamide	1.2	(0.5–3.1)	0.7
ZFTA	Trofosfamide	0.67	(0.2–2.6)	0.56
	Temozolomide	0.76	(0.2–2.6)	0.66
	Cyclophosphamide	1	(0.2–4.7)	1

*HR* hazard ratio, *95%-CI* 95% confidence-interval

### Chemotherapy salvage

To investigate whether chemotherapy was able to extend survival after STR or no resection, which normally lead to severely shortened PFS and OS, we considered all relapses with incomplete resections and looked for patients treated with chemotherapy who showed a PFS of at least 12 months after start of chemotherapy. Seven patients fitting these criteria were found, with their respective swimmer plots being shown in Fig. 1. Within these patients, three responses were still ongoing at the time of last follow-up (*Patients 3, 5, and 7*).

## Discussion

We present a heterogenous cohort of 138 patients with recurrent EPN from two multi-institutional trials and a Germany-wide registry. We analyzed the times and rates of response for different combinations and single drugs and compared the effect of chemotherapy on survival. Previous data on the systemic treatment of recurrent EPN is sparse, comprising of a mixture of clinical trials with limited case numbers and larger cohorts with diverse systemic treatments. We aim to add data from our cohort treated over the span of 20 years in Germany.

In our survival analysis we found conflicting results for the application of chemotherapy. In time-dependent Cox-regression analysis, chemotherapy showed no improvement in survival regarding PFS or OS. However, in patients with GTR/NTR, PFS could be improved by chemotherapy. We also found seven patients in whom chemotherapy was able to achieve long-term survival after subtotal or no resection. As such, a subset of patients may benefit from systemic therapy, especially if other treatment options are futile. Our findings on the lack of a general survival advantage for chemotherapy is in line with previous results [1, 2, 5].

In a small subset of seven patients with recurrences in which GTR or NTR could not be achieved—which typically show a dismal prognosis [18–20]—chemotherapy was able to induce a long-term PFS. The likelihood of this was seemingly not influenced by the molecular type, with PF-A, PF-B and ZFTA EPN falling within this subset.

Concerning responses and times to progression of specific chemotherapeutic drugs, we found sirolimus to be the most efficacious. After resections with evaluable residual tumor or no resection, we found a 50% RR and a median TTP of 11.5 months for treatment with sirolimus. However, no cases of tumor regression under treatment with sirolimus were found in our cohort, as only disease stabilization was reached. Interestingly, patients in whom sirolimus was used as a monotherapy showed an improved RR and median TTP compared to patients in whom it was used as part of a chemotherapeutic regimen of multiple drugs, mostly the RIST-protocol (NCT01467986) [21]. Pre-clinical studies on mouse ependymoma cell-lines reported that inhibition of the mTOR-pathway can induce autophagy in EPN and showed an increased survival in mice transplanted with such cell-lines when treated with sirolimus [22]. Some data also suggest an upregulation of the mTOR-pathway within EPN of the posterior fossa, with a subset of cases showing immunohistochemical staining for phosphorylated S6 [23, 24]. Clinical data on the use of sirolimus in EPN is scarce and consists of one case-report and three phase I trials not specific to EPN, reporting on a total of five recurrent EPNs, showing success in some cases [24–27]. Our findings on sirolimus are limited by the small number of applications in our cohort (n = 11). Additionally, it was applied more commonly in later recurrences, when generally MRI controls might be less frequent, which would result in a bias towards overstating the TTP. However, the markedly improved RR and TTP we found in our cohort may warrant future trials to evaluate its efficacy in recurrent EPN.

With the introduction of molecularly defined EPN types, there might be the possibility of a more individualized approach to its treatment. So far, clinical results on this matter are lacking [3, 12, 28]. To test whether any chemotherapeutic drugs were associated with better outcomes in specific molecular subgroups, we analyzed their influence on the OS in patients with PF-A or ZFTA EPN. We found no clearly significant advantages for the use of specific drugs in any subgroup. While specific drugs showed improved HRs in our analysis across different molecular types, no clear results could be found. Overall, larger case-numbers are needed to draw significant conclusions on potential benefits of specific chemotherapy-regimens in different EPN types.

Our results are limited by the non-randomized nature of the involved studies. While the HIT-REZ 97 and -2005 trials were conducted using pre-determined chemotherapy regimens at recurrence, further chemotherapies used after progression or relapse were chosen by local physicians in consultation with the trial office. The HIT-REZ registry includes only such chemotherapies chosen by local centers after consultation. This decision-making process may have led to biases on whether and which chemotherapies were chosen. For example, more intensive regimens may have been chosen in patients in whom successful long-term treatment was more likely, and therefore may show better outcomes than less intense chemotherapy chosen in palliative treatment plans. Furthermore, the measured TTP may be biased by curative vs. palliative treatment intentions, as in palliative cases and after multiple recurrences MRI and lumbar punctures may have been used more infrequently. This would lead to an overestimation of the TTP of chemotherapies especially used in patients with poor prognosis. To counteract this bias we added the DOR, which was set at 0 months for treatments if no responses than PD were found, regardless of the timing of further diagnostic tests. However, the overall low rates of response in EPN meant that the median DOR of most drugs or combinations was 0 months, making the interpretation of this parameter apart from the TTP hard.

Chemotherapy in recurrent EPN remains poorly understood. We contribute our experiences from our cohort of 138 recurrent EPN treated within Germany over the last twenty years. While we found no survival advantage for the general use of chemotherapy, we showed that in some patients, long-term tumor control via systemic treatment in absence of local therapy-options is possible. Furthermore, our data suggests that some drugs trend towards being more efficient in specific molecular types of EPN. Unfortunately, case numbers are not yet large enough to draw significant conclusions. Future pre-clinical models are offering the chance for a more individualized approach to chemotherapy and may soon influence treatment choices. While overall recurrent EPN seems to be largely resistant to chemotherapy, its use as a salvage treatment can offer improved outcome for individual patients.

## Supplementary Information

Below is the link to the electronic supplementary material.

Supplementary file1 (DOCX 98 KB)

Supplementary file2 (DOCX 50 KB)

## Data Availability

Anonymized data available upon reasonable request.

## Code availability

R code available upon reasonable request.
