# Supplementary material for: Systemic chemotherapy of pediatric recurrent ependymomas: results from the German HIT-REZ studies
Source: J Neurooncol. 2021 Oct 16;155(2):193–202. doi: 10.1007/s11060-021-03867-8 (PMC8585796; doi:10.1007/s11060-021-03867-8)
Supplement: Supplementary file 1 — Supplementary file1 (DOCX 98 KB) [file 11060_2021_3867_MOESM1_ESM.docx]

**Progression-free survival from first recurrence**


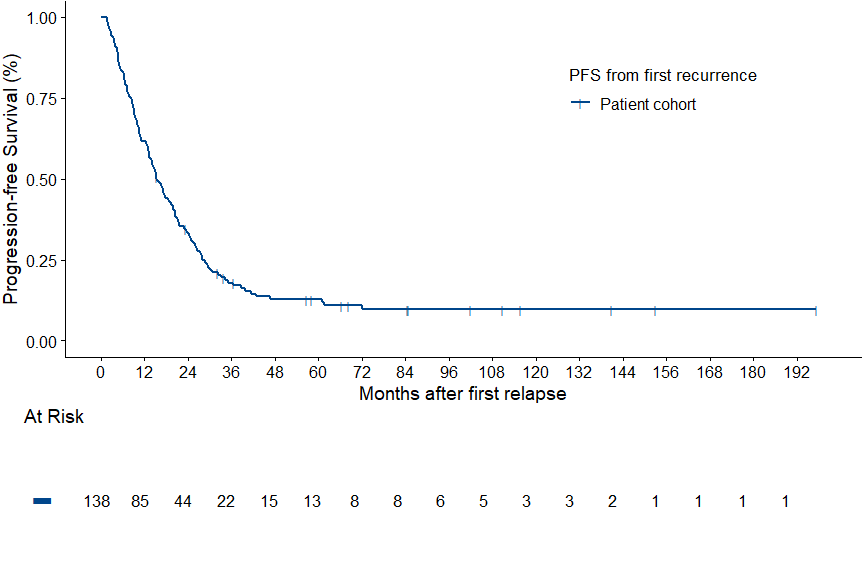


## groups median survival lower 95%-CI upper 95%-CI
## 1 Patient cohort 15.31006 13.2731 19.97536

**Overall survival after first recurrence**


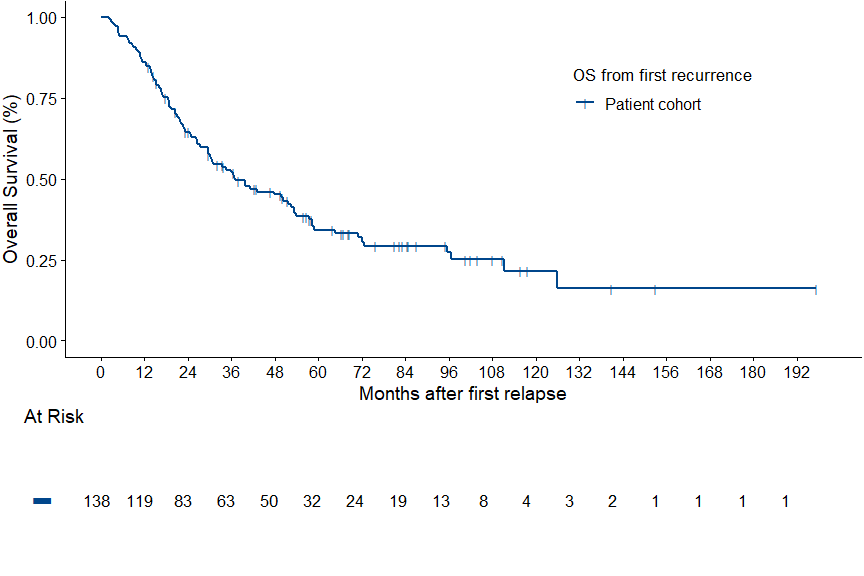


## groups median survival lower 95%-CI upper 95%-CI
## 1 Patient cohort 36.86242 29.66735 53.38809

**Progression-free survival by application of chemotherapy**


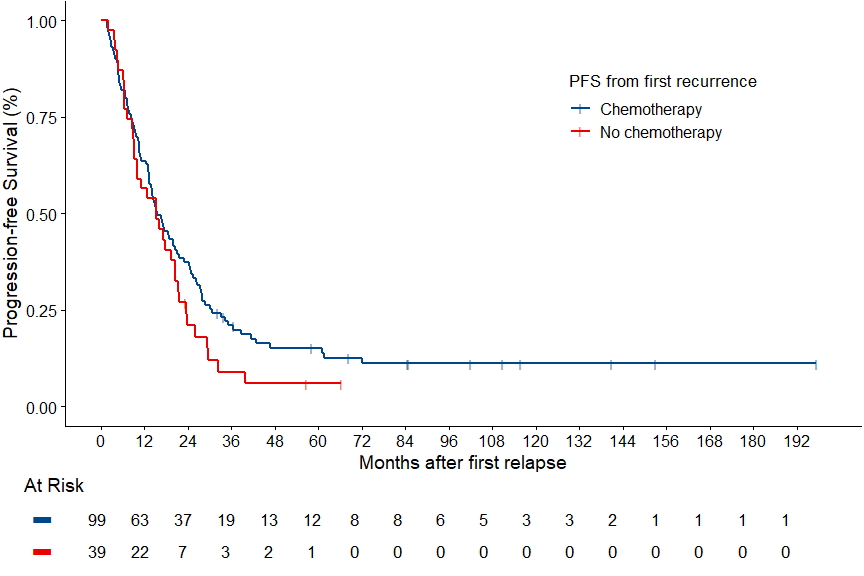


## groups median survival lower 95%-CI upper 95%-CI
## 1 Chemotherapy 15.40862 13.273101 21.48665
## 2 No chemotherapy 15.31006 9.889117 21.15811

**Overall survival by application of chemotherapy**


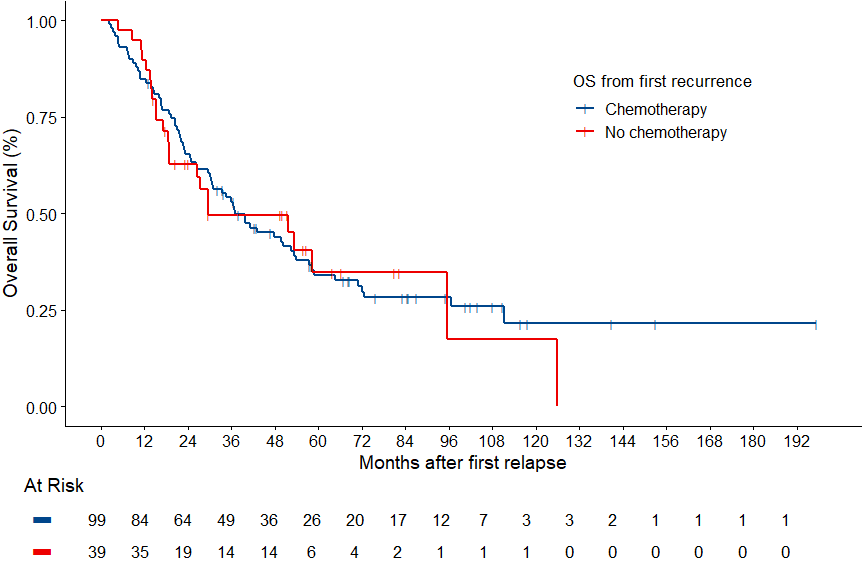


## groups median survival lower 95%-CI upper 95%-CI
## 1 Chemotherapy 36.86242 30.29158 53.71663
## 2 No chemotherapy 29.66735 18.85832 NA

**Progression-free survival of patients with GTR/NTR resection by application of chemotherapy**


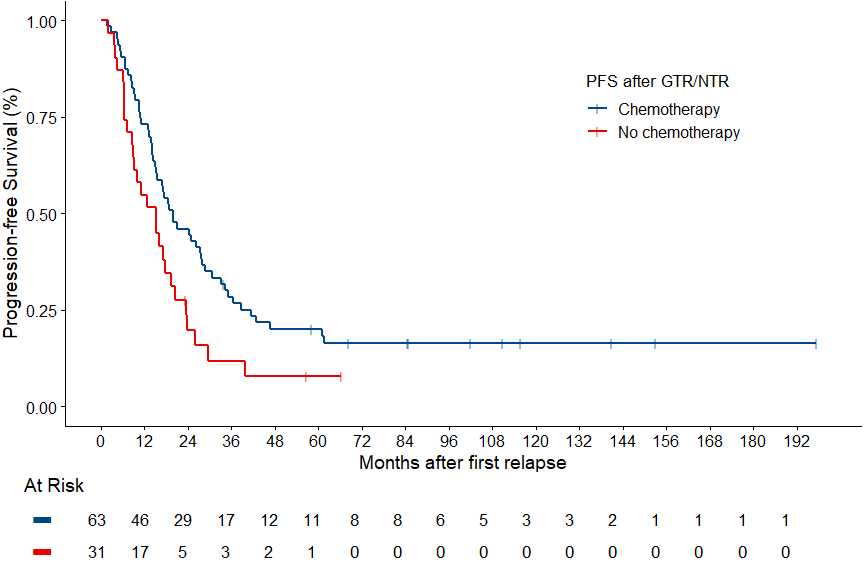

## groups median survival lower 95%-CI upper 95%-CI
## 1 Chemotherapy 19.77823 15.112936 28.84600
## 2 No chemotherapy 15.14579 8.837782 20.40246

**Overall survival of patients with GTR/NTR resection by application of chemotherapy**


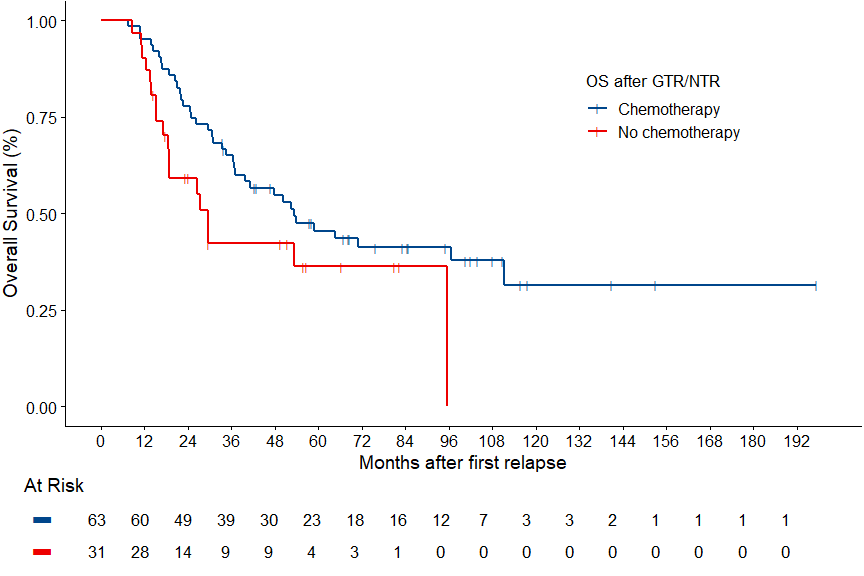

## groups median survival lower 95%-CI upper 95%-CI
## 1 Chemotherapy 53.38809 36.86242 NA
## 2 No chemotherapy 29.56879 18.75975 NA

**Progression-free survival of patients with STR / no resection by application of chemotherapy**


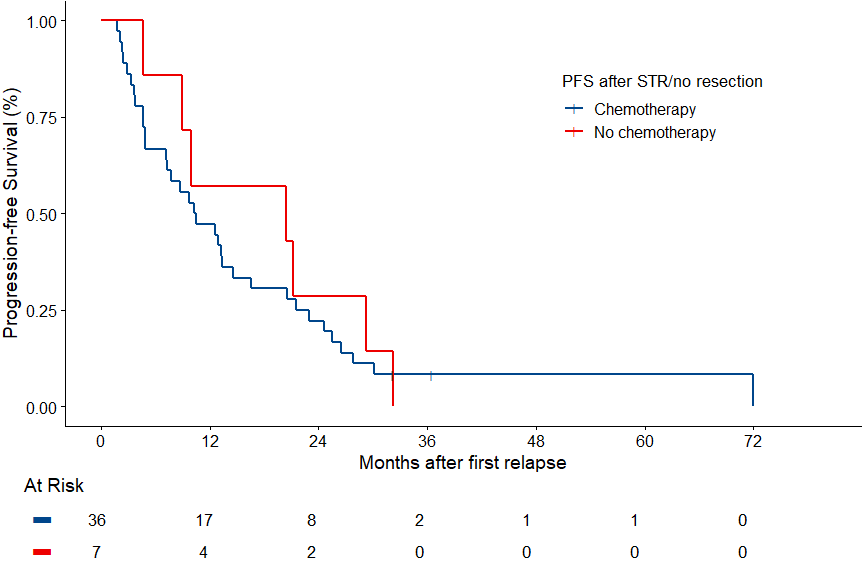

## groups median survival lower 95%-CI upper 95%-CI
## 1 Chemotherapy 10.41478 7.195072 16.52567
## 2 No chemotherapy 20.46817 9.002053 NA

**Overall survival of patients with STR / no resection by application of chemotherapy**


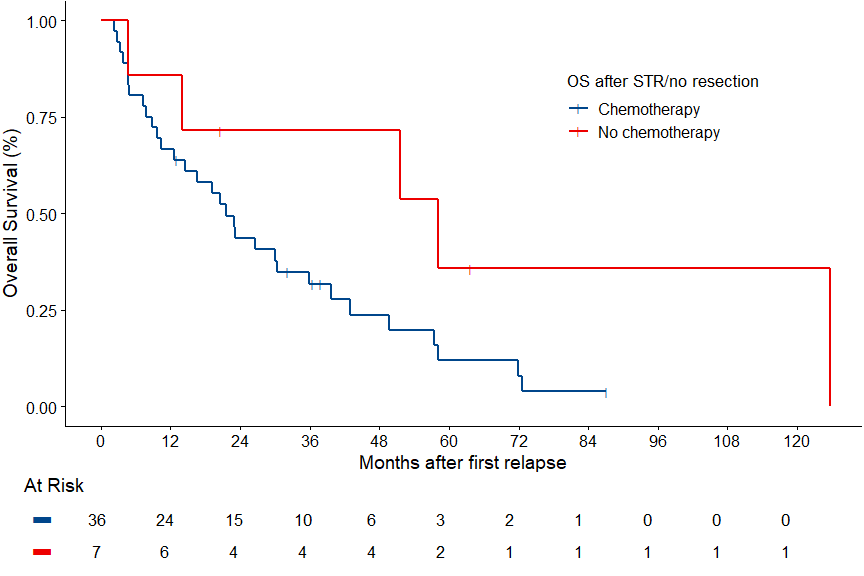

## groups median survival lower 95%-CI upper 95%-CI
## 1 Chemotherapy 21.48665 12.55031 39.68789
## 2 No chemotherapy 58.11910 14.06160 NA
