# Supplementary material for: Systemic chemotherapy of pediatric recurrent ependymomas: results from the German HIT-REZ studies
Source: J Neurooncol. 2021 Oct 16;155(2):193–202. doi: 10.1007/s11060-021-03867-8 (PMC8585796; doi:10.1007/s11060-021-03867-8)
Supplement: Supplementary file 2 — Supplementary file2 (DOCX 50 KB) [file 11060_2021_3867_MOESM2_ESM.docx]

**Supplementary Table 1** Individual chemotherapies given, combinations aswell as single-agent therapies, only evaluable relapses

| **Combination** | **n** | **CR** | **PR** | **SD** | **PD** | **ORR** | **RR** | **Median Duration of Response** | **Median Time to Progression** |
| --- | --- | --- | --- | --- | --- | --- | --- | --- | --- |
| Temozolomide | 37 | 1 | 3 | 5 | 28 | 10.8% | 24.3% | 0 (CI: 0; 0) | 2.56 (CI: 1.58; 4.7) |
| Etoposide + Trofosfamide | 21 | 0 | 1 | 7 | 13 | 4.8% | 38.1% | 0 (CI: 0; 7.4) | 3.29 (CI: 1.81; 11.44) |
| Carboplatin + Cyclophosphamide + Etoposide + Vincristine | 9 | 0 | 0 | 3 | 6 | 0% | 33.3% | 0 (CI: 0; 5.33) | 2.83 (CI: 1.89; 4.93) |
| Carboplatin + Etoposide | 9 | 0 | 1 | 3 | 5 | 11.1% | 44.4% | 0 (CI: 0; 1.64) | 8.93 (CI: 3.78; 14.22) |
| 5FU | 5 | 0 | 0 | 1 | 4 | 0% | 20% | 0 (CI: 0; 0) | 2.5 (CI: 1.35; 5.26) |
| Sirolimus | 4 | 0 | 0 | 2 | 2 | 0% | 50% | 2.46 (CI: 0; 6.29) | 14.52 (CI: 10.65; 17.68) |
| Topotecan | 4 | 0 | 0 | 0 | 4 | 0% | 0% | 0 (CI: 0; 0) | 0.55 (CI: 0.48; 0.82) |
| Carboplatin + Etoposide + Thiotepa | 2 | 0 | 0 | 1 | 1 | 0% | 50% | 3.91 (CI: 1.96; 5.86) | 7.72 (CI: 4.67; 10.78) |
| CCNU + Cisplatin | 2 | 0 | 0 | 1 | 1 | 0% | 50% | 1.89 (CI: 0.94; 2.84) | 7.25 (CI: 4.84; 9.66) |
| Celecoxib + Etoposide + Isotretionin + Temozolomide | 2 | 0 | 0 | 2 | 0 | 0% | 100% | 3.04 (CI: 1.95; 4.14) | 6.1 (CI: 3.86; 8.35) |
| Cyclophosphamide | 2 | 0 | 0 | 1 | 1 | 0% | 50% | 1.12 (CI: 0.56; 1.68) | 5.88 (CI: 4.49; 7.28) |
| Dasatinib + Irinotecan + Sirolimus + Temozolomide | 2 | 0 | 0 | 1 | 1 | 0% | 50% | 4.5 (CI: 2.25; 6.76) | 9.18 (CI: 7.02; 11.33) |
| Everolimus + Trametinib | 2 | 0 | 0 | 0 | 2 | 0% | 0% | 0 (CI: 0; 0) | 1.91 (CI: 1.26; 2.55) |
| Imatinib | 2 | 0 | 0 | 0 | 2 | 0% | 0% | 0 (CI: 0; 0) | 30.96 (CI: 28.25; 33.68) |
| Sorafenib | 2 | 0 | 0 | 0 | 2 | 0% | 0% | 0 (CI: 0; 0) | 1.52 (CI: 1.37; 1.66) |
| Trofosfamide | 2 | 0 | 0 | 1 | 1 | 0% | 50% | 0.3 (CI: 0.15; 0.44) | 5.82 (CI: 5.36; 6.28) |
| Vorinostat | 2 | 0 | 0 | 0 | 2 | 0% | 0% | 0 (CI: 0; 0) | 3.04 (CI: 2.33; 3.76) |
| ActinomycinD + Etoposide + Trofosfamide | 1 | 0 | 0 | 1 | 0 | 0% | 100% | 27.35 (CI: 27.35; 27.35) | NA (CI: NA; NA) |
| ActinomycinD + Vincristine | 1 | 0 | 0 | 0 | 1 | 0% | 0% | 0 (CI: 0; 0) | 4.18 (CI: 4.18; 4.18) |
| Afatinib | 1 | 0 | 0 | 0 | 1 | 0% | 0% | 0 (CI: 0; 0) | 1.74 (CI: 1.74; 1.74) |
| ATRA + Etoposide + Trofosfamide | 1 | 0 | 0 | 1 | 0 | 0% | 100% | 32.65 (CI: 32.65; 32.65) | 37.25 (CI: 37.25; 37.25) |
| Bevacizumab | 1 | 0 | 0 | 0 | 1 | 0% | 0% | 0 (CI: 0; 0) | 1.58 (CI: 1.58; 1.58) |
| Bevacizumab + Celecoxib + Temozolomide | 1 | 0 | 0 | 0 | 1 | 0% | 0% | 0 (CI: 0; 0) | 1.02 (CI: 1.02; 1.02) |
| Bevacizumab + Isotretionin + Tamoxifen | 1 | 0 | 0 | 0 | 1 | 0% | 0% | 0 (CI: 0; 0) | 0.92 (CI: 0.92; 0.92) |
| Celecoxib + Topotecan | 1 | 0 | 0 | 0 | 1 | 0% | 0% | 0 (CI: 0; 0) | 3.55 (CI: 3.55; 3.55) |
| Cisplatin + Etoposide + Ifosfamide | 1 | 0 | 0 | 0 | 1 | 0% | 0% | 0 (CI: 0; 0) | 15.02 (CI: 15.02; 15.02) |
| Cyclophosphamide + Etoposide | 1 | 0 | 0 | 0 | 1 | 0% | 0% | 0 (CI: 0; 0) | 1.71 (CI: 1.71; 1.71) |
| Cyclophosphamide + Mafosfamide + Vincristine | 1 | 0 | 0 | 1 | 0 | 0% | 100% | 1.74 (CI: 1.74; 1.74) | 13.18 (CI: 13.18; 13.18) |
| Etoposide | 1 | 0 | 0 | 0 | 1 | 0% | 0% | 0 (CI: 0; 0) | 1.41 (CI: 1.41; 1.41) |
| Etoposide + Hydroxycarbamide + Imatinib + Tamoxifen + Trofosfamide | 1 | 0 | 0 | 0 | 1 | 0% | 0% | 0 (CI: 0; 0) | 1.58 (CI: 1.58; 1.58) |
| Etoposide + Ifosfamide | 1 | 0 | 1 | 0 | 0 | 100% | 100% | 2.4 (CI: 2.4; 2.4) | 12.2 (CI: 12.2; 12.2) |
| Etoposide + Temozolomide + Trofosfamide | 1 | 0 | 0 | 0 | 1 | 0% | 0% | 0 (CI: 0; 0) | 13.55 (CI: 13.55; 13.55) |
| Etoposide + Topotecan + Trofosfamide | 1 | 0 | 0 | 0 | 1 | 0% | 0% | 0 (CI: 0; 0) | 1.81 (CI: 1.81; 1.81) |
| Etoposide + Trofosfamide + Valproate | 1 | 0 | 0 | 0 | 1 | 0% | 0% | 0 (CI: 0; 0) | 1.18 (CI: 1.18; 1.18) |
| Etoposide + Trofosfamide + Vinblastine | 1 | 0 | 0 | 0 | 1 | 0% | 0% | 0 (CI: 0; 0) | 5.82 (CI: 5.82; 5.82) |
| Everolimus | 1 | 0 | 0 | 0 | 1 | 0% | 0% | 0 (CI: 0; 0) | 1.15 (CI: 1.15; 1.15) |
| Hydroxycarbamide + Imatinib + Tamoxifen | 1 | 0 | 0 | 1 | 0 | 0% | 100% | 2.04 (CI: 2.04; 2.04) | 10.16 (CI: 10.16; 10.16) |
| Hydroxyurea + Valproate | 1 | 0 | 0 | 0 | 1 | 0% | 0% | 0 (CI: 0; 0) | 4.01 (CI: 4.01; 4.01) |
| Imatinib + Sirolimus + Temozolomide + Topotecan | 1 | 0 | 0 | 0 | 1 | 0% | 0% | 0 (CI: 0; 0) | 1.32 (CI: 1.32; 1.32) |
| Ipilimumab + Nivolumab | 1 | 0 | 0 | 0 | 1 | 0% | 0% | 0 (CI: 0; 0) | 8.15 (CI: 8.15; 8.15) |
| Irinotecan + Sirolimus + Sunitinib + Temozolomide | 1 | 0 | 0 | 1 | 0 | 0% | 100% | 2.56 (CI: 2.56; 2.56) | 9.57 (CI: 9.57; 9.57) |
| Palbociclib | 1 | 0 | 0 | 0 | 1 | 0% | 0% | 0 (CI: 0; 0) | 1.45 (CI: 1.45; 1.45) |
| Pembrolizumab | 1 | 0 | 0 | 0 | 1 | 0% | 0% | 0 (CI: 0; 0) | 1.28 (CI: 1.28; 1.28) |
| Sorafenib + Sunitinib + Vorinostat | 1 | 0 | 0 | 0 | 1 | 0% | 0% | 0 (CI: 0; 0) | NA (CI: NA; NA) |
| Temozolomide + Trametinib | 1 | 0 | 0 | 0 | 1 | 0% | 0% | 0 (CI: 0; 0) | 2.07 (CI: 2.07; 2.07) |
| Thalidomide | 1 | 0 | 0 | 0 | 1 | 0% | 0% | 0 (CI: 0; 0) | 1.38 (CI: 1.38; 1.38) |
| Valproate | 1 | 0 | 0 | 1 | 0 | 0% | 100% | 2.99 (CI: 2.99; 2.99) | 16.01 (CI: 16.01; 16.01) |
| Vincristine + Vorinostat | 1 | 0 | 0 | 0 | 1 | 0% | 0% | 0 (CI: 0; 0) | 5.06 (CI: 5.06; 5.06) |

**Supplementary Table 2** Individual chemotherapies given, combinations aswell as single-agent therapies, evaluable and not evaluable, RR and TTP not evaluated

| **Combination** | **n** | **CR** | **PR** | **SD** | **PD** | **Median Time to Progression** |
| --- | --- | --- | --- | --- | --- | --- |
| Temozolomide | 78 | 19 | 4 | 10 | 45 | 3.39 (CI: 1.71; 12.45) |
| Etoposide + Trofosfamide | 39 | 9 | 1 | 8 | 21 | 4.37 (CI: 1.81; 11.51) |
| Carboplatin + Cyclophosphamide + Etoposide + Vincristine | 15 | 4 | 0 | 4 | 7 | 3.29 (CI: 2.07; 9.86) |
| Carboplatin + Etoposide | 14 | 2 | 1 | 3 | 8 | 7.89 (CI: 4.5; 14.5) |
| 5FU | 6 | 1 | 0 | 1 | 4 | 3.66 (CI: 1.64; 5.15) |
| Sirolimus | 6 | 1 | 0 | 3 | 2 | 15.58 (CI: 13.45; 23.97) |
| Topotecan | 5 | 0 | 0 | 0 | 5 | 0.62 (CI: 0.49; 1.41) |
| Trofosfamide | 4 | 2 | 0 | 1 | 1 | 6.74 (CI: 5.82; 6.96) |
| Carboplatin + Etoposide + Thiotepa | 3 | 1 | 0 | 1 | 1 | 10.82 (CI: 6.22; 12.33) |
| Dasatinib + Irinotecan + Sirolimus + Temozolomide | 3 | 0 | 0 | 2 | 1 | 13.48 (CI: 9.18; 14.38) |
| Imatinib | 3 | 0 | 0 | 0 | 3 | 25.54 (CI: 14.2; 30.96) |
| Afatinib | 2 | 0 | 0 | 1 | 1 | 8.82 (CI: 5.28; 12.37) |
| Bevacizumab | 2 | 1 | 0 | 0 | 1 | 5.26 (CI: 3.42; 7.1) |
| CCNU + Cisplatin | 2 | 0 | 0 | 1 | 1 | 7.25 (CI: 4.84; 9.66) |
| Celecoxib + Etoposide + Isotretionin + Temozolomide | 2 | 0 | 0 | 2 | 0 | 6.1 (CI: 3.86; 8.35) |
| Cyclophosphamide | 2 | 0 | 0 | 1 | 1 | 5.88 (CI: 4.49; 7.28) |
| Cyclophosphamide + Melphalan | 2 | 2 | 0 | 0 | 0 | 17.38 (CI: 13.18; 21.58) |
| Etoposide + Trofosfamide + Valproate | 2 | 1 | 0 | 0 | 1 | 15.78 (CI: 8.48; 23.08) |
| Everolimus + Trametinib | 2 | 0 | 0 | 0 | 2 | 1.91 (CI: 1.26; 2.55) |
| Irinotecan + Sirolimus + Sunitinib + Temozolomide | 2 | 1 | 0 | 1 | 0 | 7.68 (CI: 6.74; 8.62) |
| Sorafenib | 2 | 0 | 0 | 0 | 2 | 1.52 (CI: 1.37; 1.66) |
| Valproate | 2 | 0 | 0 | 1 | 1 | 14.17 (CI: 13.25; 15.09) |
| Vorinostat | 2 | 0 | 0 | 0 | 2 | 3.04 (CI: 2.33; 3.76) |
| ActinomycinD | 1 | 0 | 0 | 0 | 1 | 2.27 (CI: 2.27; 2.27) |
| ActinomycinD + Etoposide + Trofosfamide | 1 | 0 | 0 | 1 | 0 | NA (CI: NA; NA) |
| ActinomycinD + Vincristine | 1 | 0 | 0 | 0 | 1 | 4.18 (CI: 4.18; 4.18) |
| ATRA + Etoposide + Trofosfamide | 1 | 0 | 0 | 1 | 0 | 37.25 (CI: 37.25; 37.25) |
| Bevacizumab + Celecoxib + Cyclophosphamide + Etoposide + Thalidomide | 1 | 0 | 0 | 0 | 1 | 2.56 (CI: 2.56; 2.56) |
| Bevacizumab + Celecoxib + Temozolomide | 1 | 0 | 0 | 0 | 1 | 1.02 (CI: 1.02; 1.02) |
| Bevacizumab + Irinotecan | 1 | 1 | 0 | 0 | 0 | 13.74 (CI: 13.74; 13.74) |
| Bevacizumab + Isotretionin + Tamoxifen | 1 | 0 | 0 | 0 | 1 | 0.92 (CI: 0.92; 0.92) |
| Bevacizumab + Sorafenib + Temozolomide | 1 | 1 | 0 | 0 | 0 | 32.68 (CI: 32.68; 32.68) |
| CCNU + Cisplatin + Vincristine | 1 | 1 | 0 | 0 | 0 | NA (CI: NA; NA) |
| Celecoxib + Topotecan | 1 | 0 | 0 | 0 | 1 | 3.55 (CI: 3.55; 3.55) |
| Cisplatin + Cyclophosphamide + Etoposide + Vincristine | 1 | 1 | 0 | 0 | 0 | 26.79 (CI: 26.79; 26.79) |
| Cisplatin + Etoposide + Ifosfamide | 1 | 0 | 0 | 0 | 1 | 15.02 (CI: 15.02; 15.02) |
| Cyclophosphamide + Etoposide | 1 | 0 | 0 | 0 | 1 | 1.71 (CI: 1.71; 1.71) |
| Cyclophosphamide + Mafosfamide + Vincristine | 1 | 0 | 0 | 1 | 0 | 13.18 (CI: 13.18; 13.18) |
| Etoposide | 1 | 0 | 0 | 0 | 1 | 1.41 (CI: 1.41; 1.41) |
| Etoposide + Hydroxycarbamide + Imatinib + Tamoxifen + Trofosfamide | 1 | 0 | 0 | 0 | 1 | 1.58 (CI: 1.58; 1.58) |
| Etoposide + Ifosfamide | 1 | 0 | 1 | 0 | 0 | 12.2 (CI: 12.2; 12.2) |
| Etoposide + Temozolomide + Trofosfamide | 1 | 0 | 0 | 0 | 1 | 13.55 (CI: 13.55; 13.55) |
| Etoposide + Topotecan + Trofosfamide | 1 | 0 | 0 | 0 | 1 | 1.81 (CI: 1.81; 1.81) |
| Etoposide + Trofosfamide + Vinblastine | 1 | 0 | 0 | 0 | 1 | 5.82 (CI: 5.82; 5.82) |
| Everolimus | 1 | 0 | 0 | 0 | 1 | 1.15 (CI: 1.15; 1.15) |
| Hydroxycarbamide + Imatinib + Tamoxifen | 1 | 0 | 0 | 1 | 0 | 10.16 (CI: 10.16; 10.16) |
| Hydroxyurea + Valproate | 1 | 0 | 0 | 0 | 1 | 4.01 (CI: 4.01; 4.01) |
| Idarubicin + Temozolomide + Topotecan + Trofosfamide | 1 | 1 | 0 | 0 | 0 | 6.08 (CI: 6.08; 6.08) |
| Idarubicin + Trofosfamide | 1 | 0 | 0 | 0 | 1 | 0.3 (CI: 0.3; 0.3) |
| Imatinib + Sirolimus + Temozolomide + Topotecan | 1 | 0 | 0 | 0 | 1 | 1.32 (CI: 1.32; 1.32) |
| Imatinib + Trofosfamide | 1 | 0 | 0 | 1 | 0 | 5.98 (CI: 5.98; 5.98) |
| Ipilimumab + Nivolumab | 1 | 0 | 0 | 0 | 1 | 8.15 (CI: 8.15; 8.15) |
| Palbociclib | 1 | 0 | 0 | 0 | 1 | 1.45 (CI: 1.45; 1.45) |
| Pembrolizumab | 1 | 0 | 0 | 0 | 1 | 1.28 (CI: 1.28; 1.28) |
| Sorafenib + Sunitinib + Vorinostat | 1 | 0 | 0 | 0 | 1 | NA (CI: NA; NA) |
| Temozolomide + Trametinib | 1 | 0 | 0 | 0 | 1 | 2.07 (CI: 2.07; 2.07) |
| Temozolomide + Vincristine | 1 | 1 | 0 | 0 | 0 | NA (CI: NA; NA) |
| Thalidomide | 1 | 0 | 0 | 0 | 1 | 1.38 (CI: 1.38; 1.38) |
| Vincristine + Vorinostat | 1 | 0 | 0 | 0 | 1 | 5.06 (CI: 5.06; 5.06) |

**Supplementary Table 3** All chemotherapy drugs used in cohort, evaluable relapses only

| **Drug** | **n** | **CR** | **PR** | **SD** | **PD** | **ORR** | **RR** | **Median Duration of Response** | **Median Time to Progression** |
| --- | --- | --- | --- | --- | --- | --- | --- | --- | --- |
| Etoposide | 54 | 0 | 3 | 18 | 33 | 5.6% | 38.9% | 0 (CI: 0; 5.04) | 3.54 (CI: 1.64; 12.45) |
| Temozolomide | 46 | 1 | 3 | 9 | 33 | 8.7% | 28.3% | 0 (CI: 0; 0.79) | 2.64 (CI: 1.59; 8.09) |
| Trofosfamide | 30 | 0 | 1 | 10 | 19 | 3.3% | 36.7% | 0 (CI: 0; 6.13) | 4 (CI: 1.81; 11.44) |
| Carboplatin | 20 | 0 | 1 | 7 | 12 | 5% | 40% | 0 (CI: 0; 4.69) | 3.93 (CI: 1.71; 12.87) |
| Cyclophosphamide | 13 | 0 | 0 | 5 | 8 | 0% | 38.5% | 0 (CI: 0; 2.24) | 3.17 (CI: 1.93; 8.97) |
| Vincristine | 12 | 0 | 0 | 4 | 8 | 0% | 33.3% | 0 (CI: 0; 2.64) | 3.29 (CI: 2.2; 7.46) |
| Sirolimus | 8 | 0 | 0 | 4 | 4 | 0% | 50% | 1.28 (CI: 0; 5.95) | 11.51 (CI: 4.22; 14) |
| Topotecan | 7 | 0 | 0 | 0 | 7 | 0% | 0% | 0 (CI: 0; 0) | 1.32 (CI: 0.55; 1.61) |
| 5FU | 5 | 0 | 0 | 1 | 4 | 0% | 20% | 0 (CI: 0; 0) | 2.5 (CI: 1.35; 5.26) |
| Imatinib | 5 | 0 | 0 | 1 | 4 | 0% | 20% | 0 (CI: 0; 0) | 10.16 (CI: 1.58; 25.54) |
| Celecoxib | 4 | 0 | 0 | 2 | 2 | 0% | 50% | 0.42 (CI: 0; 1.95) | 2.58 (CI: 1.46; 5.31) |
| Vorinostat | 4 | 0 | 0 | 0 | 4 | 0% | 0% | 0 (CI: 0; 0) | 4.47 (CI: 3.04; 4.76) |
| Bevacizumab | 3 | 0 | 0 | 0 | 3 | 0% | 0% | 0 (CI: 0; 0) | 1.02 (CI: 0.97; 1.3) |
| Cisplatin | 3 | 0 | 0 | 1 | 2 | 0% | 33.3% | 0 (CI: 0; 1.89) | 12.07 (CI: 7.25; 13.54) |
| Everolimus | 3 | 0 | 0 | 0 | 3 | 0% | 0% | 0 (CI: 0; 0) | 1.15 (CI: 0.88; 2.17) |
| Irinotecan | 3 | 0 | 0 | 2 | 1 | 0% | 66.7% | 2.56 (CI: 1.28; 5.78) | 9.57 (CI: 7.22; 11.53) |
| Isotretionin | 3 | 0 | 0 | 2 | 1 | 0% | 66.7% | 0.85 (CI: 0.42; 3.04) | 1.61 (CI: 1.27; 6.1) |
| Sorafenib | 3 | 0 | 0 | 0 | 3 | 0% | 0% | 0 (CI: 0; 0) | 1.52 (CI: 1.37; 1.66) |
| Tamoxifen | 3 | 0 | 0 | 1 | 2 | 0% | 33.3% | 0 (CI: 0; 1.02) | 1.58 (CI: 1.25; 5.87) |
| Trametinib | 3 | 0 | 0 | 0 | 3 | 0% | 0% | 0 (CI: 0; 0) | 2.07 (CI: 1.34; 2.63) |
| Valproate | 3 | 0 | 0 | 1 | 2 | 0% | 33.3% | 0 (CI: 0; 1.5) | 4.01 (CI: 2.59; 10.01) |
| ActinomycinD | 2 | 0 | 0 | 1 | 1 | 0% | 50% | 13.68 (CI: 6.84; 20.51) | 4.18 (CI: 4.18; 4.18) |
| CCNU | 2 | 0 | 0 | 1 | 1 | 0% | 50% | 1.89 (CI: 0.94; 2.84) | 7.25 (CI: 4.84; 9.66) |
| Dasatinib | 2 | 0 | 0 | 1 | 1 | 0% | 50% | 4.5 (CI: 2.25; 6.76) | 9.18 (CI: 7.02; 11.33) |
| Hydroxycarbamide | 2 | 0 | 0 | 1 | 1 | 0% | 50% | 1.02 (CI: 0.51; 1.53) | 5.87 (CI: 3.72; 8.02) |
| Ifosfamide | 2 | 0 | 1 | 0 | 1 | 50% | 50% | 1.2 (CI: 0.6; 1.8) | 13.61 (CI: 12.9; 14.32) |
| Sunitinib | 2 | 0 | 0 | 1 | 1 | 0% | 50% | 1.28 (CI: 0.64; 1.92) | 9.57 (CI: 9.57; 9.57) |
| Thiotepa | 2 | 0 | 0 | 1 | 1 | 0% | 50% | 3.91 (CI: 1.96; 5.86) | 7.72 (CI: 4.67; 10.78) |
| Afatinib | 1 | 0 | 0 | 0 | 1 | 0% | 0% | 0 (CI: 0; 0) | 1.74 (CI: 1.74; 1.74) |
| ATRA | 1 | 0 | 0 | 1 | 0 | 0% | 100% | 32.65 (CI: 32.65; 32.65) | 37.25 (CI: 37.25; 37.25) |
| Hydroxyurea | 1 | 0 | 0 | 0 | 1 | 0% | 0% | 0 (CI: 0; 0) | 4.01 (CI: 4.01; 4.01) |
| Ipilimumab | 1 | 0 | 0 | 0 | 1 | 0% | 0% | 0 (CI: 0; 0) | 8.15 (CI: 8.15; 8.15) |
| Mafosfamide | 1 | 0 | 0 | 1 | 0 | 0% | 100% | 1.74 (CI: 1.74; 1.74) | 13.18 (CI: 13.18; 13.18) |
| Nivolumab | 1 | 0 | 0 | 0 | 1 | 0% | 0% | 0 (CI: 0; 0) | 8.15 (CI: 8.15; 8.15) |
| Palbociclib | 1 | 0 | 0 | 0 | 1 | 0% | 0% | 0 (CI: 0; 0) | 1.45 (CI: 1.45; 1.45) |
| Pembrolizumab | 1 | 0 | 0 | 0 | 1 | 0% | 0% | 0 (CI: 0; 0) | 1.28 (CI: 1.28; 1.28) |
| Thalidomide | 1 | 0 | 0 | 0 | 1 | 0% | 0% | 0 (CI: 0; 0) | 1.38 (CI: 1.38; 1.38) |
| Vinblastine | 1 | 0 | 0 | 0 | 1 | 0% | 0% | 0 (CI: 0; 0) | 5.82 (CI: 5.82; 5.82) |

**Supplementary Table 4** All chemotherapy drugs used in cohort (evaluable aswell as not evaluable, RR and TTP not evaluated)

| **Drug** | **n** | **CR** | **PR** | **SD** | **PD** | **Median Time to Progression** |
| --- | --- | --- | --- | --- | --- | --- |
| Temozolomide | 92 | 23 | 4 | 15 | 50 | 4.19 (CI: 1.76; 13.04) |
| Etoposide | 87 | 18 | 3 | 20 | 46 | 5.16 (CI: 1.81; 12.92) |
| Trofosfamide | 54 | 13 | 1 | 12 | 28 | 5.36 (CI: 1.81; 11.47) |
| Carboplatin | 32 | 7 | 1 | 8 | 16 | 7.89 (CI: 2.07; 13.97) |
| Cyclophosphamide | 23 | 7 | 0 | 6 | 10 | 8.68 (CI: 2.4; 13.18) |
| Vincristine | 21 | 7 | 0 | 5 | 9 | 5.06 (CI: 2.4; 13.18) |
| Sirolimus | 12 | 2 | 0 | 6 | 4 | 13.45 (CI: 5.33; 15.43) |
| Topotecan | 9 | 1 | 0 | 0 | 8 | 1.41 (CI: 0.62; 3.55) |
| Bevacizumab | 7 | 3 | 0 | 0 | 4 | 2.56 (CI: 1.3; 11.34) |
| Imatinib | 7 | 0 | 0 | 2 | 5 | 5.98 (CI: 2.22; 17.85) |
| 5FU | 6 | 1 | 0 | 1 | 4 | 3.66 (CI: 1.64; 5.15) |
| Irinotecan | 6 | 2 | 0 | 3 | 1 | 11.53 (CI: 6.74; 13.68) |
| Celecoxib | 5 | 0 | 0 | 2 | 3 | 2.56 (CI: 1.61; 3.55) |
| Cisplatin | 5 | 2 | 0 | 1 | 2 | 13.54 (CI: 9.66; 17.96) |
| Valproate | 5 | 1 | 0 | 1 | 3 | 12.33 (CI: 4.01; 16.01) |
| Sorafenib | 4 | 1 | 0 | 0 | 3 | 1.81 (CI: 1.52; 17.25) |
| Vorinostat | 4 | 0 | 0 | 0 | 4 | 4.47 (CI: 3.04; 4.76) |
| ActinomycinD | 3 | 0 | 0 | 1 | 2 | 3.22 (CI: 2.75; 3.7) |
| CCNU | 3 | 1 | 0 | 1 | 1 | 7.25 (CI: 4.84; 9.66) |
| Dasatinib | 3 | 0 | 0 | 2 | 1 | 13.48 (CI: 9.18; 14.38) |
| Everolimus | 3 | 0 | 0 | 0 | 3 | 1.15 (CI: 0.88; 2.17) |
| Isotretionin | 3 | 0 | 0 | 2 | 1 | 1.61 (CI: 1.27; 6.1) |
| Sunitinib | 3 | 1 | 0 | 1 | 1 | 7.68 (CI: 6.74; 8.62) |
| Tamoxifen | 3 | 0 | 0 | 1 | 2 | 1.58 (CI: 1.25; 5.87) |
| Thiotepa | 3 | 1 | 0 | 1 | 1 | 10.82 (CI: 6.22; 12.33) |
| Trametinib | 3 | 0 | 0 | 0 | 3 | 2.07 (CI: 1.34; 2.63) |
| Afatinib | 2 | 0 | 0 | 1 | 1 | 8.82 (CI: 5.28; 12.37) |
| Hydroxycarbamide | 2 | 0 | 0 | 1 | 1 | 5.87 (CI: 3.72; 8.02) |
| Idarubicin | 2 | 1 | 0 | 0 | 1 | 3.19 (CI: 1.75; 4.64) |
| Ifosfamide | 2 | 0 | 1 | 0 | 1 | 13.61 (CI: 12.9; 14.32) |
| Melphalan | 2 | 2 | 0 | 0 | 0 | 17.38 (CI: 13.18; 21.58) |
| Thalidomide | 2 | 0 | 0 | 0 | 2 | 1.97 (CI: 1.67; 2.26) |
| ATRA | 1 | 0 | 0 | 1 | 0 | 37.25 (CI: 37.25; 37.25) |
| Hydroxyurea | 1 | 0 | 0 | 0 | 1 | 4.01 (CI: 4.01; 4.01) |
| Ipilimumab | 1 | 0 | 0 | 0 | 1 | 8.15 (CI: 8.15; 8.15) |
| Mafosfamide | 1 | 0 | 0 | 1 | 0 | 13.18 (CI: 13.18; 13.18) |
| Nivolumab | 1 | 0 | 0 | 0 | 1 | 8.15 (CI: 8.15; 8.15) |
| Palbociclib | 1 | 0 | 0 | 0 | 1 | 1.45 (CI: 1.45; 1.45) |
| Pembrolizumab | 1 | 0 | 0 | 0 | 1 | 1.28 (CI: 1.28; 1.28) |
| Vinblastine | 1 | 0 | 0 | 0 | 1 | 5.82 (CI: 5.82; 5.82) |
